# Becoming in Resistance: The (Un)Creative Relation Between Non-heterosexual Identity and Psychological Suffering

**DOI:** 10.3389/fpsyg.2020.502755

**Published:** 2020-09-15

**Authors:** Sebastián Collado, Carolina Besoain

**Affiliations:** Facultad de Psicología, Universidad Alberto Hurtado, Santiago, Chile

**Keywords:** identity, self, creativity, queer-feminism, dialogism, LGBTIQ^∗^, non-heterosexual identities

## Abstract

This article aims at theorizing a creative and processual theory of non-heterosexual identity. It will be argued that, so far, scholars have tended to theorize non-heterosexual identity from a monologic perspective, which establishes one-sidedly a casual and/or unproblematic relation between the emergence of forms of psychological suffering and the development of a non-heterosexual identity. Although it must be recognized that such a claim is important at a political level, at a subjective level, it leaves non-heterosexual people destined to be flooded by distressing and painful emotional states. To counter monologism within theorisations of non-heterosexual identity development, without ignoring the negative impacts of heteronormativity, it will be argued that non-heterosexual identity needs to be theorized (1) as part of a creative process situated in a specific sociohistorical context marked by heteronormativity, (2) as part of a situated process that produces and never ceases to produce multiple effects (self-states), which are unified to create an identity, and (3) as part of a situated process of creation that can be artificially transformed through art. These are the three claims that will move forward the argument of this article.

## Introduction

Since the introduction of the concept of homophobia (Weinberg, 1972), a relationship between non-heterosexual identities and different forms of psychological suffering has been amply demonstrated (Meyer, 2003; Herek, 2004; King et al., 2008; Lewis, 2009; Cook et al., 2014; Berg et al., 2015; Barrientos et al., 2016; Semlyen et al., 2016). Some studies have even shown that suicide rates are up to seven times higher within non-heterosexual people than heterosexual people (Tomicic et al., 2016). Worryingly, when comparing recent studies on the relationship between psychological suffering and non-heterosexual identities internationally, the situation does not seem to improve (Siqueira et al., 2009; Ghorayeb and Dalgalarrondo, 2011; Smith, 2011; Barrientos and Cárdenas, 2013; Pérez, 2014; Meyer, 2015; Flores, 2019). Thus, the evidence appears clear: the development of a non-heterosexual identity implicates negative material consequences at the level of subjective experience of non-heterosexual people, namely, the emergence of different forms of psychological suffering.

Since the depathologization of homosexuality, different psychosocial and socially sensitive theories have been developed to explain the problematic relation between non-heterosexual identity and psychological suffering (Cass, 1979; Troiden, 1989; Butler, 1997; Meyer, 2003). Despite their differences, all theories recognize that the emergence of psychological suffering in non-heterosexual people is related to the difficulty of developing a non-heterosexual identity in societies marked by pervasive homophobic violence and heteronormativity (see Warner, 1991; Bilodeau and Renn, 2005). However, the specific processes through which a non-heterosexual person is affected by homophobic violence and heteronormativity in terms of her identity development have been addressed in very different ways. In general terms, these theories can be divided between *synthesizing* and *critical* theories of non-heterosexual identities.

Drawing upon the identity theory of Erikson (1956, 1980), *synthesizing* theories were developed mostly in the 70’ and 80’ by authors such as Cass (1979, 1984) and Troiden (1989). In these theories, the emergence of psychological suffering is related to the capacity of the non-heterosexual person to arrive to an identity synthesis state after identity development. Although these theories have been widely used in empirical research and clinical practice, in the last three decades they have been deeply criticized, and have thus lost their heuristic value (see Kenneady and Oswalt, 2014; Ferdoush, 2016, for a discussion).

On the *critical* side, the performative theory of identity developed by the queer-feminist scholar Butler (1990, 1997) became a groundbreaking alternative to explain the relationship between non-heterosexual identities and psychological suffering. Drawing upon Freud’s (1915/1991) and Lacan’s (1977) psychoanalytic theories of human development and Foucault’s (1978) ideas of discourse, in which sexual difference is the cornerstone of sociocultural organization, Butler (1997) indicates that the heteronormative organization of society implies a process of individual identifications, which inevitably “spawn forms of melancholy” (p. 144). This is valid both for heterosexual and non-heterosexual people, however, for non-heterosexual people, due to historically situated heteronormative social norms the disavowal and unspeakability of their identifications “can achieve suicidal proportions” (p. 148). Although Butler’s (1997) alternative theoretical explanation is of major importance at a political level, allowing the exposure of unjust power relations, paradoxically, on the level of subjective experience, it has left non-heterosexual people somehow predestined to experience only one possible emotional state after developing a non-heterosexual identity: namely, melancholy.

This tendency to *condemn* the non-heterosexual person to only one emotional state after identity development is not only present in Butler’s (1990; 1997), but is typical of some brunches of poststructuralist queer-feminism (see also Sedwick, 1985, 1990; Jagose, 2001): an intellectual trend that draws upon Foucault’s genealogical analysis to theorize non-heterosexual identities. Whilst we acknowledge the political value of queer-feminist theory in the tradition of Butler (1990, 1997), we consider it problematic when it comes to the conceptualization of non-heterosexual identity, for it proposes a conceptualization in which identity is mostly understood as a suspicious constant recreation (and not creation) of the heteronormative norm (see Sedwick, 2003, for a discussion of the hermeneutic of suspicion of queer-feminist theory). In fact, one important implication of Butler’s (1997) theory of identity development is the assumption that the relation between psychological suffering and non-heterosexual identity does not need to be theorized, because there is an obvious causal relation between non-heterosexual identity and psychological suffering mediated by the norm (see Haraway, 1988, for more elaboration of this point).

However, if one wants to better understand not only how the norm is recreated in its Butlerian fashion, but also how it is agentively appropriated and transformed by non-heterosexual people, we consider it necessary to theorize non-heterosexual identity and not only the norm. In this regard, we argue that while there are norms that constrain non-heterosexual identities, there is also the potential for developments that defy an inevitable result such as Butler’s (1997) melancholy or other forms of psychological suffering. To support such an argument, we claim that non-heterosexual identity needs to be theorized (1) as part of a creative process situated in a specific sociohistorical context marked by heteronormativity, (2) as part of a situated process that produces and never ceases to produce multiple effects (self-states), which are unified to create an identity, and (3) as part of a situated process of creation that can be artificially transformed through the contemporary artistic practices of performance art. These three claims will move the argument of this article forward.

In the light of worrying evidence regarding the relation between psychological suffering and non-heterosexual identity, it is important for psychology in general, and therapeutic practice in particular, to better understand how the process of developing a non-heterosexual identity works in the context of heteronormativity. Although the more obvious result of such a process is the emergence of different forms of psychological suffering, a less monologic theory of non-heterosexual identity, that is, a theory that goes beyond identity as solely the effect of the norm and focuses also on processual and creative aspects of non-heterosexual identity development, can find creative spaces for potential developments that do not fall (only) into melancholy or other forms of psychological suffering.

There have been many theoretical approaches to the self as a creative process of becoming (see Bergson, 1946/2007; Deleuze and Guattari, 2000). However, there are not so many that specifically theorize identity and/or a unification state of the self as an important part of the creative process of self-becoming. Therefore, methodologically, this article will build an argument to support the three claims made above with the help of theories of becoming oneself, which understand the self as a creative and dialogical movement between self-multiplicity (non-identity) and self-unification (identity). When we use the terms creative and dialogical, our methodological proposal is of course inspired by the work of Bakhtin (1934–1935/1981, 1952–1953/1986) on dialogism and language, but also by the feminist and relational reading of the development of the self offered by the psychoanalytic scholar Benjamin (1988, 2018), in which the self is understood as a life-long process of creative negotiation of different and paradoxical self-states that moves dialectically between self-multiplicity (non-identity) and oneness (identity).

Considering the epistemologies that inform our methodological approach, we have selected the theories of creative becoming developed by Ricoeur, 1970 and Winnicott (1971) to support our first two claims. We suggest, perhaps arguably for some readers (cf. Strawson, 2004; Phelan, 2005), that these theories can be read both dialogically and creatively (see also Leiman, 1992; Priel, 1999; Collington, 2001; Ellis and Stam, 2010; Glaveanu, 2010). However, since these authors do not theorize the process of becoming oneself in the context of heteronormativity, we will also bring queer-feminist authors into the discussion such as Butler (2001, 2004, 2005) and Benjamin (2018), who place Ricoeur, 1970 and Winnicott (1971) in this specific context. To support our third claim, we will work with the theoretical approach to contemporary art practices pioneered by Rancière (2009a,b, 2013), which places the self and its political/subjective transformation at the center.

Concretely, in the first section the argument will focus mainly on the concept of narrative identity developed by Ricoeur (1992) in *Oneself as Another*, which, as we will demonstrate, was prefigured by a dialogical and creative stance of his preceding works. Since for Ricoeur (1992) heteronormativity is problematically invisible, his theoretical proposal will be complemented by the later works of Butler (2001, 2004, 2005), which for the most part, in opposition to her first theories of non-heterosexual identity, abandon her hermeneutics of suspicion and get much closer to the hermeneutics of trust developed by Ricoeur (1970) (see also Sedwick, 2003). In the second section, the argument will move to Winnicott’s (1971) relational psychodynamic theorization of the self and the relationship that his theory has both with creativity and psychological suffering. Since Winnicott (1971) does not theorize on non-heterosexual identity, we will draw upon the work of queer-feminist scholars to bring his theory of the self in line with the research object of this article. With the help of Rancière (2009a,b, 2013) art theory, in the fourth section it will be argued that institutionalized contemporary art practices such as performance art can be theorized as a creative space in which non-heterosexual people can explore playfully and transform their identities. All sections of the article aim to support the three claims made above. As it will be seen in the concluding section, when non-heterosexual identity is understood non-monologically as a provisional result of a historically situated and life-long creative process that moves between unification and multiplicity, the apparently causal relationship between non-heterosexual identity and psychological suffering becomes disrupted.

## Non-Heterosexual Identity Beyond Norms

As it has already been suggested, the first theories of non-heterosexual identity developed by Butler (1990, 1997) present some theoretical flaws. The first problem in Butler’s (1990) theories can be found in her seminal work *Gender Trouble*, which by giving too much attention to newness and resistance, tends to leave undertheorized those aspects of the self that, despite its unessential nature, tend to remain stable. The second problem can be found in *The Psychic Life of Power*, in which, in order to address the stable aspects of the self, Butler (1997) makes reference to Freud’s (1915/1991) theory of melancholy, concluding that under heteronormative social conditions non-heterosexual identity (and actually any identity) is destined to be flooded by melancholy. In this reading, heteronormativity is seen as a rather static structure with which both non-heterosexual and heterosexual people identify. It is important to consider that for Butler (1990, 1997), drawing upon Lacan (1953/2012), Lacan (1977), identity is, at least theoretically, always non-essential and, somehow, also nonexistent. However, for Butler (1997) identity becomes a rather fixed intrapsychic structure via libidinized identifications with the norm. As the reader will see, this changes in Butler (2001, 2004, 2005) later works in which this intrapsychic structure is no longer seen so problematically. For instance, in *Giving an Account of Oneself*, identity is seen more as a necessary creative, even artistic, process for personal survival achieved through a self-narrative: “no one can live in a radically non-narratable world or survive a radically non-narratable life” (p. 34).

In *Oneself as Another*, Ricoeur (1992) elaborates a theory of a socio-historically situated and non-essential self – that is, a self as “an event in the world” (p. 30), which, unlike other non-essentialist accounts found in Lacan (1953/2012), Lacan (1977), the early works of Butler (1990, 1997), and Foucault (1978), does not consider personal identity to be as problematic, or at least not for the same reasons. The first theoretical movement that allows Ricoeur (1992) to be, paradoxically, “non-essential” without dismissing identity is to separate two different but interdependent aspects of the self: sameness or idem-identity, and selfhood or ipse-identity. Thus, for the philosopher, identity is paradoxically both change and continuity, both newness and stability. Before continuing with the question of identity, it is important to understand some historical epistemological tendencies of Ricoeur (1992). This will help the reader to understand why this article considers that Ricoeur (1992) can be interpreted dialogically and creatively, and also why such an interpretation supports some aspects of the first two claims presented in the introductory section: (1) identity as part of a situated process, and (2) identity as a unification of the multiple effects of that process.

In Ricoeur’s (1970) critique of Freud’s (1915/1991) theory of the self, he identified some epistemological ambiguities in Freud’s psychoanalytic approach: self as a biopsychological structure vs. self as linguistic process. Later, criticizing Lacan (1953/2012), Ricoeur (1970) argued that the French psychoanalyst erroneously solved Freud’s (1915/1991) epistemological confusions by imposing the norms of the linguistic structuralism of Saussure (1933/1945), Jakobson (1960), and Levi-Strauss (1962) upon the unconscious and conscious self, a theoretical movement that Ricoeur (1970) clearly rejects (see also Simms, 2007; Bussachi, 2016).

In later works, Ricoeur (1975, 1978) clarifies his theory of language that, 30 years before *Oneself as Another*, separates it from structuralist understandings of the self. In short, Ricoeur (1975, 1978) uses Benveniste’s (1966) theory of language (sociolinguistics) rather than the ones proposed by structuralism. For Benveniste (1966), as well as for Ricoeur (1985, 1992), the self develops and never ceases to develop in and through discourse, that is, through the concrete use of language which is always living and embodied in a specific “public time and space” (Ricoeur, 1992, p. 50), and not as an effect of the ahistorical norms of language (see also Billig, 1997). Hence, language is understood as both the materiality and the action through which the self comes into being (Ricoeur, 1992, pp. 33–39).

Using Benveniste’s (1966) approach to language, it appears that in Ricoeur (1970, 1975, 1978, 1985) work, there exists a displacement from semiotics to semantics, and from the instance of the letter to the instance of discourse (see Ricoeur, 1985, p. 53). It was exactly this displacement that would allow Ricoeur (1985) to argue in *Time and Narrative* that the experience of human time, that is, the experience of having a life that develops throughout a temporal dimension, is narratively created through the use of language (pp. 61–64). Hence, without a narrative – however partial and provisional this narrative may be – the self would be deprived of sociohistorical existence. It is this idea of deprivation of sociohistorical existence due to the absence of personal and social narratives that Butler (2001, 2004, 2005) recognizes in her works after the *Psychic Life of Power*.

In *Giving an Account of Oneself*, it is easy to observe that Butler (2001, 2005) is critical of narrative theories that impose on subjects the obligation to deliver a narrative of themselves. For Butler (2001, 2005), some branches of contemporary psychology have been particularly harmful because of their tendency not only to impose the necessity of a narrative but also the insistence that it must be a coherent one. However, as the reader will see later, taking into account the dialogic possibilities of contemporary narratives that Ricoeur (1985) identifies in *Time and Narrative*, it could be argued that what Butler (2001, 2005) criticizes are the monologic heteronormative narratives imposed upon subjects, which do not offer them many possibilities for being. On the contrary, they render invisible the diverse forms that a historical embodied life could take.

Although for Butler, 1990 language has played a major role in her theories of identity and subjectivity, it is not easy to follow her epistemological path regarding language, as it can be done with Ricoeur (1970, 1975, 1978, 1985). Nevertheless, what can be observed is her tendency to move back and forth from a more structuralist approach to language, in which identity is only the (subjugated) effect of the constraining norms of language, and a more dialogic approach, in which “the signifiers of identity are not structurally determined in advance” (Butler, 1997, p. 104).

This rather confusing movement between different approaches to language ceases in *Giving an Account of Oneself*. What changes in this work is the importance that Butler (2001, 2005) ascribes to historically situated intersubjective relations mediated and made possible when one is not only controlled and constrained by language, but uses it to give a narrative account of oneself. By doing this, Butler (2001, 2005) comes much closer to Ricoeur’s (1992) understanding of the relation between language and identity, in which personal identity develops within a “dialogic skeleton of highly diversified interpersonal exchanges” (p. 44). Nevertheless, what differentiates Butler (2001) from Ricoeur (1992) is her argument that these interpersonal exchanges within which any possible identity develops must be placed in a “scene of address” (Butler, 2001, p. 33), which is predefined by social norms that regulate the directions that a self-narrative can take. Although something similar to a scene of address – a “context of interlocution” (Ricoeur, 1992, p. 192) – is vaguely addressed by Ricoeur (1992), the concrete material conditions that constitute a scene of address remains somewhat unclear. As it was claimed in the introductory section, for a theory of non-heterosexual identity, it is not enough to say that identity is a narrative process. Identity is a narrative process that takes place in the context of heteronormativity, thus, the vagueness of Ricoeur (1992) regarding the context in which identity develops is unacceptable and needs to be corrected.

Drawing upon Benveniste (1966), for Ricoeur (1992), one’s own identity is always performed by an “I–You” (p. 41), there is never an “I” alone. Hence, the very emergence of a sense of being something such as an “I” that *owns* an identity is preceded by a “You” that demands its presence. Considering the heteronormative scene of address identified by Butler (2001, 2004, 2005), this demand never takes place in a vacuum. This means that the “I” is not called to become whatever it wants in terms of identity, rather, there are heteronormative norms, that is, historical monologic narratives regarding sexuality, that would need to be accepted first, if the “I” were to become recognized by the “You” as a valid “I.” This certainly leaves non-heterosexual people in a dangerous position. However, by focusing too narrowly on the monologic narratives that constrain identity, Butler (2001, 2005) overlooks the complex processes that make possible the very existence of this identity, and also in turn the possibilities that could be opened up if one understood these processes dialogically.

According to Ricoeur (1992), the process through which personal identity develops is called the “self.” The self occurs and never ceases to occur between two poles that Ricoeur (1992) calls idem-identity and ipse-identity, or sameness and selfhood. The pole of sameness (idem-identity) is associated with that part of the self that remains, more or less constant and is recognizable by the “I” and others as being the same throughout time (e.g., one’s own character). Nevertheless, the problem of sameness is that it “cover[s] over the innovation which preceded, even to the point of abolishing the latter” (p. 121). The sameness pole of the self are “acquired habits” (p. 122) that have been rehearsed and performed over and over through language-mediated intersubjective relations within a dialogic discursive field, that is, within a specific historic and linguistically organized sociocultural context populated by multiple ideologized perspectives (see also Bakhtin, 1934–1935/1981, pp. 270–272). Because the pole of sameness develops within a dialogic discursive field, the sameness of the self can be theorized as a narrative (linguistic) and provisional unification of multiple experienced perspectives of the self about itself. Nevertheless, although these perspectives are experienced individually by the self, they always carry a historically situated ideological weight.

This idea of the self as narratively becoming within a dialogical discursive field presented in *Oneself as Another* was already developed in *Time and Narrative* through Ricoeur’s (1985) analysis of Bakhtin’s (1934–1935/1981) dialogic novel. The introduction of dialogism into Ricoeur’s (1985) theory of narrativity had an impact on his later theory of narrative identity. It is, indeed, dialogism in narrative identity that allows us to suggest that non-heterosexual identity does not need to fall, at least not always, into the pervasive monologism imposed by heteronormativity.

From our interpretation of Ricoeur (1985), narratives are material and situated human actions – “configuring act[s]” (p. 61) – which are not constrained by the norms of language but by preceding narratives. As Ricoeur (1985) indicates a narrative is that particular “poetic composition” (p. 94) which, as a cultural resource, is in charge of creating artistically (artificially) the social and individual experience of human time (see also Arfuch, 2010). It is precisely the experience of time that transforms a biological organism into a historical human being with a life that has been meaningfully lived and that can be remembered. This is what leads Ricoeur (1985) to state that a personal narrative gives meaning to one’s own remembered life “by elevating meaningless life to a meaningful work by the grace of art” (p. 80). It is important, though, to consider that narratives as sociohistorical and situated artistic actions have changed throughout history.

Before the dialogic narrative theorized by Bakhtin (1934–1935/1981), Ricoeur (1985) indicates that traditional European narratives were rather “monologic” (p. 96). This means that the narrator was the organizing principle, the owner of the experience of time. On the contrary, Bakhtin (1934–1935/1981) dialogic narrative breaks the hierarchy in which the narrator always has the last word. This means that the dialogical narrative is open to the multiple voices – ideologized perspectives, in Bakhtin (1934–1935/1981) terminology (see also Larrain and Haye, 2019) – that constitute the dialogic discursive field in which every human life unfolds. This also has consequences in terms of time. There is no more a singular experience of Time, but rather multiple perspectives of an experienced time that cannot be detached from the material lives of the characters. In sum, what the dialogic narrative as cultural resource achieves is to question the ways in which narratives have historically helped human beings to create the experience of time, exposing that singular Time was rather a consequence of “centripetal forces” (Bakhtin, 1934–1935/1981, p. 271), that is, of material social struggles, rather than the natural order of things.

We argue that Butler (1990, 1997) earlier works on non-heterosexual identity offer a theory which focuses one-sidedly on the heteronormative centripetal forces of discourse, forces that try to monologize the dialogism of the discursive field of life. From a dialogic perspective, however, while there are centripetal forces, there are also “centrifugal forces” (Bakhtin, 1934–1935/1981, p. 272) within a dialogic discursive field. This means that in a discursively organized heteronormative society, while intersubjective relations tend to recreate the heteronormative norm, they also inevitably create new norms and transform old ones. This can be seen in some countries since the 70′, for instance, in the creation of new meanings regarding non-heterosexuality that have slowly become accepted ideologized perspectives regarding human sexuality. This is what Butler (2001, 2005) starts recognizing in her later works. A dialogic discursive field can seem to be totally unified by heteronormative centripetal forces, however, as Bakhtin (1934–1935/1981) states, “alongside verbal-ideological centralization and unification, the uninterrupted processes of decentralization and disunification go forward (…) the centripetal forces of the life of language, embodied in a unitary language operate in the midst of heteroglossia” (p. 272).

Drawing upon a dialogic perspective, in *Oneself as Another* Ricoeur (1992) argues that the sameness pole of the self (idem-identity) develops processually within a discursive field constituted by an enormous number of historically contingent “preferences, evaluations, and estimations” (p. 122), that is, an enormous number of ideologized perspectives, that are created, interchanged, and stabilized through language-mediated human relations. Through a complex process of linguistic praxis with many others (and not only with the Other), these multiple ideologized perspectives are internalized by the self. It is this process of internalization – a process always inaugurated by others – that paradoxically “annuls the initial effect of otherness” (Ricoeur, 1992, p. 122), transforming the multiple ideologized perspectives encountered *outside* into the base semiotic (linguistic) material needed to develop what lives *inside*, namely, sameness (idem-identity).

From a dialogical paradigm, the possibility of encountering ideologized perspectives that despise non-heterosexual people in a heteronormative society is rather high, however, it cannot be assumed as the only possibility. This can be seen in qualitative investigations that, in recent years, have tried to counter the one-sided portrayal of non-heterosexual people as eternal victims of homophobic violence (see Hammack and Cohler, 2009; Davis, 2015; Sala and De la Mata Benítez, 2016). These investigations show an inner dialogical movement of the self in which heteronormative ideologized perspectives encounter non-heteronormative perspectives producing a sort of intrasubjective friction. Thus, we argue that the self is never totally occupied by only one ideologized perspective, not even at the level of one single internalized linguistic sign.

By interpreting Ricoeur’s (1992) work dialogically, the possibility of moving from monologism to dialogism can be found in the other pole of identity, namely, in ipse-identity. Ipse-identity is what Ricoeur (1992) calls the pole of innovations, that part of the process of becoming oneself which is always open for transformation. Ipse-identity defies the sameness of the self without destroying it, but by creatively integrating into sameness (idem-identity) new ideologized perspectives experienced by the self. How does one move from idem-identity to ipse-identity? Narrative identity is the linguistic device/action through which the sameness of the self can be creatively questioned and re-composed (p. 123). In sum, narrative identity, when understood as a linguistic creative action, has the capacity, on the one hand, to exhibit that the sameness of the self is more a contraction of many internalized ideologized perspectives rather than a solid and stable substratum, and on the other hand, to create artificially, even fictionally, new versions of sameness (see also Butler, 2001, p. 26).

From a perspective that takes seriously the pervasive destructiveness of the centripetal forces of heteronormativity, what remains a critical point in Ricoeur (1992) is that the creativity that narrative identity requires to move from monologism to dialogism is taken for granted. For Ricoeur (1992) creativity is neither problematized nor theorized. But, is this really so? Is creativity for non-heterosexual people as available as it is for people who conform the norms of heteronormativity? These are questions that will be addressed in the next section. For now, with the help of Ricoeur (1985, 1992) and Butler (1997, 2001, 2004, 2005), it is possible to re-state the first two claims made in the introductory section: non-heterosexual identity can be conceptualized as (1) part of a process situated in a context marked by monologic heteronormativity and (2) as part of a process though which new ideologized perspectives experienced and internalized by the self can be unified into sameness. Is that process a creative one? To answer this question, let us move to the next section to explore creativity and its relation to non-heterosexual identity.

## Creativity

For Winnicott (1971), creativity plays an essential role when it comes to the “search of the self” (p. 71). Although he did not conceptualize the relevance of dialogism in his theory, later authors have argued that Winnicott’s (1971) work on the self implies a dialogic understanding of intersubjectivity in which creativity both sociogenetically develops and is supported by others (Leiman, 1992; Priel, 1999; Virno, 2002; Glaveanu, 2010). Creativity in Winnicott’s (1971) theory of the self is essential for it to “remain true to itself” (Ruti, 2011, p. 360). Similar to Ricoeur’s (1992) conceptualization of personal identity, this *true self* does not imply a solid self, but rather a self in an state of creativity, of being alive, which for Winnicott (1971) is essential for the creation of a human life (and not organic life) that is worth living. But, before engaging with the relation between the self and creativity, the integration of *play* into his theory of the self needs to be addressed.

In *Playing and Reality*, Winnicott (1971) establishes that “only in playing, the child or adult is free to be creative” (p. 71). In his work more broadly, the capacity to play creatively with one’s own life, that is, to endure and embrace paradoxical self-states (see also Benjamin, 2018), is closely linked to subjective well-being and the reduction of psychological suffering. Winnicott (1971) indicates this clearly in his statement that a creative life gives the individual a sense that life is “worth living” (p. 87), and that, on the other hand, an uncreative and compliant life, experienced as “something to be fitted in” (p. 87), creates the feeling “that nothing matters and that life is not worth living” (p. 87). Furthermore, Winnicott (1971) indicates that the absence of play, which results in the absence of creativity, even makes some individuals think that “suicide is of small importance” (p. 92), because what play enhances is the capacity not only to produce creative external things but also to tolerate paradoxical and painful self-states in the “moment-by-moment living” (p. 92.) (see also Winnicott, 1959).

What is interesting in Winnicott’s (1971) work is that play in childhood, and every cultural experience in adulthood, is never an *internal experience*, but a liminal action that takes place first in “the overlap of two play areas” (p. 72) and later becomes *internal*. This resembles Ricoeur’s (1992) idea that the internalization of ideologized perspectives are first experienced in intersubjective relations, and later become the semiotic materials for creating one’s own identity (see also Billig, 1997). The relevance of this is that play, and consequently also creativity, can be seen not as a solipsistic activity but as an intersubjective experience that can only occur in, and is intrinsically dependent on, the environment in which individuals develop. Using more dialogic terminology, dependent on the semiotically organized discursive field in which play takes place (see also Leiman, 1992).

Benjamin (2018), in her feminist reading of Winnicott (1971), interprets play as an action that creates a liminal space – a ‘Third Space’– in which multiple and, at times, contradictory perspectives of reality have a place. In play, body and mind can move relatively freely between alternative perspectives of reality, without getting stuck in one perspective of it. Thus, the kind of creativity that play fosters is one that helps people to “entertain incompatible versions of what is going on” (p. 145), that is, to be able to hold paradoxical perspectives without necessarily getting rid of one or synthetizing them. However, authors such as Burack (1995) and Goldner (2003) make us aware that play is regulated by historically contingent gendered and sexualized norms. Hence, as it was argued in the preceding section, play and creativity within heteronormativity cannot be taken for granted. This will be addressed again later.

Regarding the self, Winnicott (1971) understands it as a “never-ending and essentially unsuccessful” (p. 73) process of becoming. From our interpretation of Winnicott (1971), the self is an embodied socio-psychic process of becoming that develops in specific time-spaces shared with others. This means that the self as process is constituted by a more or less unintegrated accumulation of multiple self-states, that is, of multiple perspectives of reality experienced by the self. These multiple perspectives are, first, experienced in intersubjective relations and, later, internalized. After internalization, these multiple perspectives of reality are unified, producing the provisional effect of the self as a unity.

Furthermore, intersubjective relations can be nourishing, but they can also be radically harming, constituting what Winnicott (1971) calls “pathological communities” (p. 93). In pathological communities, play as a nourishing action that fosters creativity cannot take place, which implies, following Benjamin (2018), that only one perspective of reality, that is, only one perspective of the self, can exist. This resembles Goldner’s (2003) queer-feminist reading of Winnicott (1971), which argues that a pathological community such as a heteronormative society reproduces compulsorily the monologic narrative of “two mutually exclusive sexes” (Goldner, 2003, p. 127). This implicates that non-heterosexual aspects of identity (non-heterosexual self-states), because of their incongruence with “normative femininity or masculinity, would have to be foreclosed, disavowed, displaced, disguised, projected, or otherwise evacuated” (p. 128).

In contrast to other approaches to psychological suffering, for Winnicott (1971), since the self is basically a movement between unintegration and unity, a back and forth between multiplicity and oneness, psychological suffering does not relate to the unintegrated nature of the self, but rather to the way in which the environment imposes an experience of this unintegration of multiplicity as a failure. Here, Winnicott (1971) is not arguing that psychological suffering would fade away if persons experienced themselves in a constant state of unintegration. On the contrary, Winnicott (1971) considers that a minimal state of unity of the self, that is, a minimal state of sameness, is indispensable if one is to experience life as worth living.

The paradox is that for Winnicott (1971), the state of unity required for living is not a given and remains unfinished until the end of life. This means that unity needs to be constantly maintained, and the capacity for this maintenance, which is enabled by creativity, can only be experienced in (protected) states of unintegration, that is, in play, in which the paradoxical and contradicting multiplicities of the self have the right to exist. In more dialogical words, without experiencing the dialogism of the self, the self cannot develop the necessary creativity to hold through unification its own multiplicity. This leads to a situation in which some internalized self-states are lived as not-self-states, remaining alienated/alienating.

Up to this point, it can be observed that creativity relates to the capacity of the individual to hold her own contradicting and paradoxical self-states through the creation of a provisional state of unity. However, creativity is not an individual feature, but is enabled by an environment that allows persons to experience their multiplicity as a success and not as a failure. In this regard, with Goldner (2003), it became clear that in a heteronormative society non-heterosexual people are, somehow, forced to live an uncreative life, even to the point of being pushed to evacuate their own embodied self. Does there for non-heterosexual people, then, remain any space for creativity? Are we not going back to Butler’s (1997) theory of inevitable melancholy? Here, Benjamin’s (2018) feminist re-reading of Winnicott (1971) becomes relevant.

Benjamin (2018) indicates that methodologically traditional psychoanalysis, even in the tradition of Winnicott (1971), has refused to use discursive genres other than verbal self-narratives and verbal free association. In contrast, Benjamin (2018) considers that contemporary art practices such as performance art have a lot to teach psychoanalysts, when it comes to the enhancement of creativity, specially the creativity of people whose creative potential has been limited by heteronormative relations (see also Benjamin, 1988). The specificity of performance art will be addressed in greater detail in the next section. For now, it can be said that what Benjamin (2018) sees in performance art is the possibility of opening intersubjective spaces of play beyond traditional therapeutic settings in which verbal and non-verbal aspects of the self can be explored. Furthermore, as it is well recognized by Benjamin (2018), when approaching performance art as a process of creation and not only as a product, intersubjective verbal and non-verbal patterns of discursive communication can be recreated, and new possibilities can be improvised. According to Benjamin (2018), these features of performance art need to be considered if heteronormative power relations are to be transformed at a psychological level.

The discussion on creativity led in this section gives us a good basis for exploring how a non-heterosexual self can be theorized as a *possibly creative* process, and non-heterosexual identity as a needed provisional unification of multiple self-states reached in that process. We say *possibly creative* because in a society in which monologic heteronormativity is reproduced over and over through intersubjective relations, it is highly unlikely that internalized non-heterosexual self-states can be experienced as owned-self-states or integrated into that provisional unification that is identity. Creativity as the unifying psychological function of the self depends on experiencing multiple self-states as possible versions of it. Nevertheless, in opposition to other theories of non-heterosexual identity, from a Winnicottian perspective the self is a life-long unfinished process, which is always open to transformation through the enhancement of creativity. As it was suggested by Benjamin (2018), performance art can be seen as a space of play beyond traditional therapy, in which creativity can be stimulated. To comprehend better Benjamin’s (2018) claim, let us explore performance art in greater detail.

## Transformation Through Art

In this section, we will focus on moving the argument to the third claim made in the introductory section: that non-heterosexual identity can be transformed through the contemporary art practice of performance art.

Despite the importance of creativity in Winnicott’s (1971) work, he makes a clear distinction between his understandings of creativity as creative living and art as an institutionalized activity that produces final and coherent results. Since the self is thought of as a process characterized by dialogism, Winnicott’s (1971) creativity relates to the capacity to hold multiple self-states (experienced reality perspectives) on a daily basis, rather than to the capacity to produce results. Therefore, he indicates that the artists analyzed by Freud (1910/1957) are not necessarily creative, at least not under his understanding of creativity as a daily phenomenon (see Winnicott, 1971/2015, p. 93; Milner, 1987/2002, p. 201). It must be stated, however, that Winnicott’s (1971) concept of art as an institutionalized activity that only produces results is only one possible reading of art.

Winnicott’s (1971) understanding of art refers to what Rancière (2009b) calls a “representational regime of art” (p. 7). Based on a Kantian tradition, the representational regime of art is part of a philosophical movement in which art is reduced to its capacity to produce beautiful forms that can transcend the mundane aspects of social and personal life. Therefore, this philosophy of art focuses on establishing the norms that regulate what can be defined as art and the ways in which artistic products must be presented to an audience in terms of form. From such a conceptualization, the processual and non-technical aspects of art practices are not theorized.

Rancière (2009b) places in modernity a philosophical revolution within art theory that he calls the “aesthetic regime of art” (p. 46). In the aesthetic regime, the creative process of making art gains a significant place. This does not mean that the mastery of artistic techniques gets lost, but that art practices are not limited to technical acquisition. Instead, artistic creation looks for the redistribution and reorganization of a personal/social life that is embedded in an ideologized and semiotically organized discursive field. This can be clearly seen in *The Aesthetic Unconscious* where Rancière (2009a) indicates that what artists do is to take signifying/ideologized elements (signs) of their social and personal reality and recompose them artistically in a novel manner (see p. 34). Hence, art is not a matter of transcending the mundane, but of transforming the mundane social/personal reality that occurs while artists (and, eventually, non-artists) play creatively with formal and content aspects of their social/personal lives. Based on this conception of art, artistic production is understood as a provisional gesture of unification, which results from a creative process of play that artificially cuts out a piece of the experienced world to explore and transform it (see Rancière, 2009b, pp. 33–35).

According to Rancière (2013), among different art practices, performance art has become a particularly useful tool for playfully exploring, questioning, and transforming the self at a psychological level (see also Rancière, 2009a).

An important feature of performance art is that it tries to intentionally “suspend the normal coordinates of sensory experience” (Rancière, 2009b, p. 25) by playing with ideologized elements of social/personal reality. This means that performance art aims at questioning, through artistic means, what is regarded as normal, exposing the historical and political forces behind the normal coordinates of experienced reality. Therefore, for oppressed communities such as non-heterosexual people, performance art has proven to be a useful institutionalized activity for exploring their own oppression in terms of its causes, but also in terms of the possibilities of experiencing life beyond that oppression (see Heddon, 2006, for a discussion of the importance of performance art in the context of heteronormative oppression).

In this context, experiencing life beyond oppression means to transform the monologic experiential repertoire of life. In dialogical terms: to move from one experienced perspective of reality to multiple experienced perspectives of it. There are good theoretical reasons to think that for a person who has experienced reality too often from the perspective of a survivor of heteronormative violence, to open life to other lived experiences can be a transformational process at an individual psychological level. However, performance art does not only look for the multiplication of experience, but also for a provisional unification of the new gained perspectives of reality.

Performance art can be understood as an embodied research project, in which artists engage emotionally, cognitively, and behaviorally. In fact, according to Rancière (2009b), the specificity of performance art is that “thoughts, practices, and affects are [not] instituted and assigned a territory or a specific object” (p. 5), and instead the limits between these psychological aspects of the self become blurred. During the creative process, the embodied research project of performance art looks intentionally to gain new and multiple emotional/cognitive/behavioral perspectives of reality, for instance, by reading, discussing, enacting scenes, inquiring into one’s own biography, experiencing new emotions, etc. Furthermore, in performance art new perspectives are not experienced once, but are repetitively rehearsed and presented. This gives performance practitioners the possibility of inhabiting and internalizing those new perspectives.

However, since performance art normally finishes with a public showing, the new gained perspectives of reality are unified into a provisional performative gesture that can be shared with others. As it was suggested in the preceding sections, this unification process is crucial in terms of creativity. Multiplication of experienced perspectives just for the sake of it does not necessarily enhance creativity – what does enhance it, though, is the process of unifying all those perhaps paradoxical and contradicting perspectives of reality. In Winnicottian terms: the process of learning of how to hold multiplicity without getting lost in it. Or, as Ricoeur (1992) puts is: the process of achieving creatively “discordant concordance” (p. 141) in one’s own narrative.

In the introductory section, it was argued that non-heterosexual identity can be artificially transformed through performance art. As highlighted in this section, in performance art the non-heterosexual self can engage in an embodied research process in which the heteronormative organization of the discursive field of life is artistically/artificially suspended. During the creation process, the non-heterosexual self has the opportunity to experience repetitively reality from perspectives that were restricted before due to the heteronormative organization of the discursive field, in which the self and its identity develop (see also Billig, 1997). If, as demonstrated in the first sections, non-heterosexual identity is theorized as a provisional unification of self-sates experienced throughout the process of living in a society marked by monologic heteronormativity, there are good reasons to think that gaining new self-states, that is, new experienced perspectives of reality, can transform that provisional unification. Performance art offers a playful space in which the process of non-heterosexual identity development can be artistically/artificially unfolded so that sedimented self-states can be revisited and questioned. However, as it was seen above, the aim of performance art is not to dissolve that provisional unification, but to fabricate a new provisional unification artistically/artificially, integrating self-states that could not be integrated before.

## Conclusion

This article aimed at theorizing non-heterosexual identity from a less monologic perspective than it is in existing literature. Preceding theorizations have argued one-sidedly that non-heterosexual identities are, somehow, destined to develop different forms of psychological suffering under heteronormative life conditions (see Butler, 1997). From this perspective, non-heterosexual identity does not need to be theorized because the problem is located only at the level of social norms. Non-heterosexual identity appears to be simply a causal effect of heteronormativity. To counter this monologic and non-agentive approach, we claimed that non-heterosexual identity would need to be theorized (1) as part of a creative process situated in a specific sociohistorical context marked by heteronormativity, (2) as part of a situated process that produces multiple effects (self-states), which are over and over unified to create an identity, and (3) as part of a situated process of creation that can be artistically/artificially transformed through performance art.

Throughout the article we discussed theories of identity/unification that support these three claims. Through a dialogic interpretation of Ricoeur, 1970, it became clear that identity is not only sameness. Identity as sameness is the narrative contraction of the dialogism of self-processes, which never ceases to be open to the dialogism of discursive life: what Ricoeur (1992) calls ipse-identity. The self as process happens within a discursive field populated by “preferences, evaluations, and estimations” (p. 122), that through internalization become the semiotic (linguistic) materials through which identity as sameness can be developed. The action of narrative identity has the capacity to open identity as sameness to identity as ipse, so that the sameness of the self can be creatively recomposed. However, with Butler (2001, 2004, 2005) it could be suggested that under heteronormative social conditions the basic requirement for the action of narrative identity, namely, creativity, cannot be taken for granted.

In the second section, with Winnicott (1971), it could be seen that heteronormativity impedes the development of creativity through preventing the full experience of non-heterosexual self-states. Creativity was conceptualized in this section as the psychological function that makes possible the provisional unification of the self, namely, identity. However, the creative potential of a non-heterosexual self can only develop if all of her self-states can be, first, fully experienced and, later, internalized, otherwise the non-heterosexual self-states cannot be appropriated as semiotic materials for new unifications of the self. From this perspective, creativity remains a potential possibility, a possibility that can be enhanced by bringing the non-heterosexual person to the creative terrain of play. At the end of this section, performance art was theorized as an institutionalized practice, in which play, and consequently creativity, can take place and identity can be transformed.

In the third section, performance art was theorized as a transformative practice beyond traditional therapy, in which non-heterosexual people can fully experience self-states that are normally prohibited under heteronormative life conditions. This can be done because the creative process of performance art can artistically/artificially suspend the normal coordinates of social and personal life, allowing the non-heterosexual person to experience reality playfully from new and different perspectives. Through rehearsals and repetitions, these new perspectives can become internalized semiotic materials with which the non-heterosexual person can start playing, so that she tries out different provisional unifications of herself.

To theorize non-heterosexual identity as the unification of multiple self-states that happens and never ceases to happen in the context of a dialogic process that we call the self, allows us to suggest that the emergence of forms psychological suffering is a possibility but not a destiny. From a dialogic perspective of non-heterosexual identity, it can be theorized that while a non-heterosexual person experiences the prohibition of her non-heterosexual self-states, she is, at least potentially, open to fully experiencing, inhabiting, and internalizing these self-states. As it was indicated in the first section, despite the strength of the centripetal forces of heteronormativity, life always unfolds in the midst of heteroglossia. This means that identarian monologism can be overcome by moving the non-heterosexual person from monologism to dialogism, so that she can start experiencing life from multiple perspectives beyond hegemonic heteronormativity. Hence, dialogically speaking, melancholy and other forms of psychological suffering are possible self-states, but not the only ones.

Our proposal tries to counter theories which conceptualize non-heterosexual identity as monologic. However, our theoretical account presents some limitations. Although we want to overcome monologism within non-heterosexual identity theorizing by showing that non-heterosexual identity is not condemned to be constituted by only one self-state, we recognize that the available empirical evidence tends to undermine our theory. This could be due to the tendency of empirical research to intentionally look for the negative effects of heteronormativity on non-heterosexual identities. While this has been valuable politically, it also serves to erase resilience and resistance strategies, which are not only present on an unconscious and individual level, but are collective, material practices which need to be acknowledged, researched, and resourced. This could be particularly productive in more deprived contexts outside the Global North in which practices such as individual therapy is not within everybody’s reach, and also in radically homophobic contexts in which the possibility of identifying openly as non-heterosexual is not a real option.

## Author Contributions

SC led the research, drafted the first version of the manuscript, and reviewed the final one. CB co-led the research and drafted the second version of the manuscript. Both authors contributed to the article and approved the submitted version.

## Conflict of Interest

The authors declare that the research was conducted in the absence of any commercial or financial relationships that could be construed as a potential conflict of interest.
